# Rapid assay to assess colonization patterns following in-vivo probiotic ingestion

**DOI:** 10.1186/1756-0500-6-252

**Published:** 2013-07-05

**Authors:** Jacinta M Tobin, Suzanne M Garland, Susan E Jacobs, Marie Pirotta, Sepehr N Tabrizi

**Affiliations:** 1 Northwest Academic Centre University of Melbourne, Sunshine Hospital, St Albans, VIC 3021, Australia; 2 Department of Microbiology and Infectious Diseases, The Royal Women’s Hospital, Parkville, VIC, Australia; 3 Department of Obstetrics and Gynaecology, University of Melbourne, Parkville, VIC 3052, Australia; 4 Murdoch Childrens Research Institute, Parkville, VIC 3052, Australia; 5 Neonatal Services, The Royal Women’s Hospital, Parkville, VIC, Australia; 6 Department of General Practice, University of Melbourne, Parkville, VIC 3052, Australia

**Keywords:** Probiotics, qPCR, Preterm infant, Micro biome

## Abstract

**Background:**

Colonization of the intestine with some microorganisms has been shown to have beneficial health effects. The association of bacteria with its human host starts soon after birth; however in infants born prematurely establishment of normal intestinal flora is interrupted with colonization with potential pathogenic organisms Probiotic supplementation may therefore be beneficial to the health of preterm infants. As most probiotic organisms are difficult to culture, confirmation of their colonization after supplementation is difficult. In this study, rapid qPCR assays for detection of presence of probiotic species in the intestine by faecal sampling is described in both preterm infant and adult participants.

**Findings:**

Probiotic colonization was determined using qPCR directed at amplification of organisms present in the ingested probiotic *Streptococcus thermophilus, Bifidobacterium animalis subsp. lactis* and *B. longum subsp. infantis.* Overall, differential detection of probiotic strains in faeces were found between adult and preterm infants, with 50% of infants continuing to shed at least two probiotic strains three weeks after probiotic ingestion had ceased.

**Conclusions:**

This study demonstrated rapid assessment of the preterm infant gut for colonization with probiotic strains using real-time PCR*.* This method would be of great importance in studies of probiotics in prevention of diseases and adverse clinical outcomes.

## Findings

The intestinal micro biota contains a diverse range of bacterial species [1]. The bacterial community has also been shown to have a symbiotic relationship with their human host and is beneficial to human health [2,3]. This association starts soon after birth [4]. *Bifidobacterium* species (*B*. spp), commonly found in the intestine of healthy infants, may influence postnatal immune development, including susceptibility to sepsis and allergic diseases [5]. In preterm infants, the development of the intestinal micro biome is affected by the neonatal intensive care unit environment, with decreased microbial diversity, including colonization with potential pathogens and association with late-onset sepsis and necrotizing enterocolitis [6,7]. Supplementation with appropriate probiotic strains including *B*.spp may therefore be beneficial to the health of preterm infants, subject to confirmation of detection in fecal output and possible colonization. However, probiotic organisms are mostly fastidious anaerobes and difficult to culture using conventional techniques, as well as difficult to speciate, being closely related to other naturally occurring species [8]. Rapid, sensitive, molecular tests can provide a faster and more accurate method to confirm micro biota colonization [9,10]. In this study, changes in detection of *in-vivo* probiotic ingested organisms were measured using quantitative real-time PCR directed to specific detection of the three organisms present in the formulation.

Following approval by the Royal Women’s Hospital Human Research and Ethics Committees, 12 preterm infants born below 32 weeks gestation, weighing less than 1500 g and seven healthy adult volunteers were recruited to participate. Written informed consent for participation in the study was obtained from participants or, in cases of infants from their parent or guardian. The probiotic formulation, ABC Dophilus for Infants®, (Solgar, New York, USA) containing 2 × 10^8^*Bifidobacterium longum subsp. infantis*, 2.3 × 10^8^*B. animalis subsp. lactis* and 2.3 × 10^8^*Streptococcus thermophilus* per gram was ingested by adult participants (15 g or 10^10^ organisms) and 6 preterm infants (1.5 g or 10^9^ organisms) for seven days, while six control infants were fed routinely with their mother’s expressed breast milk.

Specimens for assay were collected by rotating a flocked swab in feces, and then rotated in 400 μL of phosphate buffered saline (PBS). Fecal samples were collected prior to and at completion of probiotic ingestion, then weekly up to 4 weeks and stored at −80°C until analyzed. Two hundred μl of cell suspensions were extracted using MagNA Pure LC system (Roche Diagnostics, Branchburg, NJ) with the associated DNA Isolation Kit I protocol. DNA was eluted in a final volume of 100 μL of MagNA Pure Elution Buffer (Roche Diagnostics). Extracted DNA was tested by three quantitative real-time PCR (qPCR) assays for detection of *B. longum subsp. infantis, S. thermophilus* and *B. lactis*. These assays are not strain specific and will only detect the respective species within the sample being tested.

Each qPCRs was performed using the Light Cycler 480 real-time instrument (Roche Diagnostics), with each reaction comprised of a 20 μl volume, containing 1x Light Cycler 480 probes master mix (Roche Diagnostics), 1.0 μM each previously published specie-specific primers [11-13] (Table 1), 200 nM each probe (Table 1), and 5 μl of DNA template. All qPCR reactions had the same cycling parameters of 95°C for 10 minutes followed by 50 cycles of 95°C for 10 seconds and 60°C for 55 seconds. Data for each sample were plotted as a derivative of fluorescence versus temperature. All samples which yielded linear increases in their fluorescence readings relative to the negative control sample were considered positive. Strict procedures avoiding specimen contamination and carryover were followed.

**Table 1:** PCR primer and probes sequences utilized in the study

	**Sequence (5′-3′)**^**a**^	
**Target**	**Primer/probe**	**Ref**
*B. animalis subsp. lactis*	F: GTGGAGACACGGTTTCCC	[13]
	R: CACACCACACAATCCAATAC	[13]
	P: FAM-TTCACAGGTGGTGCATGGTCGT BHQ1	This paper
*B. longum subsp. infantis*	F: TTCCAGTTGATCGCATGGTC	[12]
	R: GGAAACCCCATCTCTGGGAT	[12]
	P: CY5-TCAAgCCCAggTAAggTTCTTCgC BHQ3	This paper
*S. thermophilus*	F: TTATTTGAAAGGGGCAATTGCT	[11] Modified
	R: GTGAACTTTCCACTCTCACAC	[11] Modified
	P: CY5-ACTACAAGATGGACCTGCGT BHQ3	This paper

^a^Nucleotide bases among the primers and probes that are underlined represent locked nucleic acid bases. F, forward primer; R, reverse primer; P, probe; FAM, 6-carboxyfluorescein; Cy5, cyanine 5; BHQ, Black Hole Quencher.

**Figure 1:**
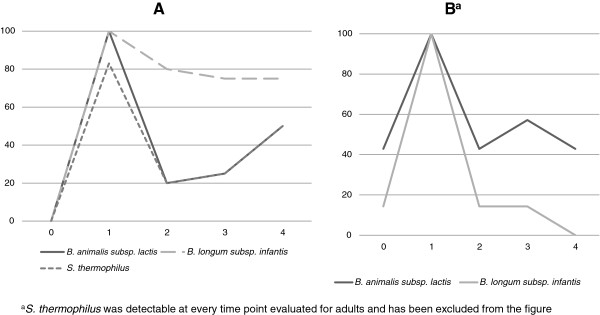
**Percentage of samples positive at each sampling time for each probiotic strain from participants who ingested the ABC Dophilus. A)** Infant **B)** Adult participants.

All three probiotic species were detectable in 83% (5/6) of infants who ingested probiotics by Week 1 (Figure 1A). None of the three probiotic species were detected in the 12 preterm infants’ feces at Week 0 and subsequently none of the 6 control infants had the probiotic strains detected. *B. longum subsp. infantis* continued to be detected in 75% of the infants four weeks after probiotic ingestion had ceased.

In the adult group, *S. thermophilus* was present before and throughout the intervention in all participants, due to presence of this organism in readily available cheese and yoghurts. *B. longum subsp. infantis* was detected in feces of all 7 adult participants at Week 1, but not four weeks after probiotic ingestion had ceased, whereas *B. animalis subsp. lactis* was detectable in up to 40% of adults, 4 weeks post probiotic ingestion (Figure 1B).

It is important to test a pre-supplementation sample to ensure the subject is not positive for the probiotic organisms prior to its administration, as it is possible for gut micro biota to already contain the organism [14]. This study demonstrated rapid assessment using real-time PCR of preterm infant gut for colonization of ingested probiotic strains*.* This method would be a valuable tool in studies evaluating probiotics in prevention of diseases and adverse clinical outcomes [15].

## Competing interests

In the past 5 years none of the authors have received reimbursements, fees, funding or salary from any organization that may in any way gain or lose financially from the publication of this manuscript either now or in the future. In addition there is no non-financial competing interest to declare in relation to this manuscript.

## Authors’ contributions

ST, SG, JT, MP and SJ conceived and participated in the design of the study. ST carried out qPCR as well as drafted the manuscript. SG, JT, SJ, MP assisted in preparation of the manuscript. JT and SG coordinated the specimen collection. All authors read and approved the final manuscript.
